# Fatigue in adults with primary antiphospholipid syndrome: findings from a mixed-methods study

**DOI:** 10.1177/0961203320928421

**Published:** 2020-06-05

**Authors:** Lindsay M Bearne, Julie Bieles, Sofia Georgopoulou, Josie Andrews, Amy Tully, Katrine Stolarchuk-Prowting, Tom Williamson, Beatriz Santana Suarez, Louise Nel, David D’Cruz, Heidi Lempp

**Affiliations:** 1 Department of Population Health Sciences, King’s College London, London, UK; 2 Centre for Rheumatic Diseases, Department of Inflammation Biology, King’s College London, London, UK; 3 Guys and St Thomas Hospitals NHS Foundation Trust, London, UK; 4 Department of Inflammation Biology, King’s College London, London, UK

**Keywords:** Antiphospholipid syndrome, fatigue, mixed methods

## Abstract

**Objective:**

This study aimed to explore the experience and impact of fatigue in adults with primary antiphospholipid syndrome (pAPS).

**Methods:**

This sequential, explanatory mixed-methods study enrolled adults with a six-month or more history of pAPS. Consenting participants completed the Functional Assessment of Chronic Illness Therapy–Fatigue subscale (FS), Multi-Dimensional Perceived Social Support Scale, Patient Health Questionnaire (PHQ9), Pittsburgh Sleep Quality Index (PSQI), International Physical Activity Questionnaire (IPAQMETS). Relationships between FS and other variables were explored with multiple linear regression. Interviews were conducted with a subgroup of participants, and the data were analysed thematically.

**Results:**

A total of 103 participants were recruited (*M*_age_ = 50.3 years; standard deviation = 10.1 years; 18 males). Of these, 62% reported severe fatigue. Greater fatigue was associated with lower mood, physical inactivity, poorer sleep quality and lower perceived social support. The best-fit model explained 56% of the variance in FS (adjusted *R*^2^ = 0.560, F(3, 74) = 33.65, *p* > 0.001) and included PHQ9 and IPAQMETS as significant predictors, and PSQI as a non-significant predictor. Twenty participants completed interviews. Three key themes were identified: characteristics of fatigue, impact on life and coping strategies.

**Conclusion:**

Fatigue was a common symptom of pAPS and challenging to manage. Other factors, particularly mood and physical activity, influenced fatigue. Evidence-based self-management interventions are needed.

## Introduction

Antiphospholipid syndrome (APS) is an autoimmune prothrombotic disorder which occurs as a distinct clinical syndrome in isolation (primary APS (pAPS)) or with other rheumatic and musculoskeletal diseases (RMDs) such as systemic lupus erythematosus (SLE).^1^ The incidence of APS is approximately 5/100,000 people annually, and the prevalence is 40–50/100,000 people.^1^ More women than men are affected (female:male ratio 5:1), and there is no racial prevalence for pAPS.^2,3^ Its clinical spectrum includes venous and/or arterial thromboses and pregnancy morbidity in the presence of antiphospholipid antibodies (e.g. lupus anticoagulant, anticardiolipin antibodies and anti-β2 glycoprotein-I antibodies). People with pAPS also experience other symptoms, such as fatigue and low mood, and have difficulty keeping physically active.^4,5^

Chronic fatigue is an unpleasant, abnormal or excessive whole-body tiredness, disproportionate to or unrelated to activity or exertion and present for more than one month. It is not relieved easily by sleep or rest.^6,7^ Fatigue is a common symptom of RMDs and adversely affects an individual’s health status, as well as physical and social function. Studies in RMDs (e.g. rheumatoid arthritis (RA)^8^ and SLE^9^) and other long-term conditions (e.g. multiple sclerosis^10^) have highlighted the impact and importance of fatigue.^11–13^

The aetiology of fatigue is poorly understood. Theoretical models propose fatigue is a multi-causal, multidimensional symptom where disease-related (e.g. disease activity), cognitive/emotional (e.g. mood), physical and social factors (e.g. physical activity and social support) interact with each other.^14–16^ In other RMDs, aspects outside the direct disease effects account for more of the variation of fatigue than condition-related features.^15,17,18^

For this study, key factors which influence fatigue in other RMDs were identified. Low mood is common in people with RMDs and is consistently correlated with higher fatigue.^16,18,19^ Low mood is also linked with sleep disturbance, and evidence suggests that poor sleep quality influences fatigue levels.^15,20,21^ Physical activity tends to be low in people with RMDs,^22,23^ and physical inactivity is associated with higher fatigue.^15,24,25^ This relationship may be mediated by mood, sleep or obesity.^15,24,25^ Social support from family, friends or significant others (e.g. health-care professionals) may also influence fatigue.^16,26^

Few studies have explored the experience and impact of fatigue in people with pAPS, and it is rarely acknowledged in evidence-based management recommendations.^27^ To inform the development of non-pharmacological interventions, a mixed-methods approach is needed that comprises quantitative data to investigate the relationships between fatigue and key variables and qualitative data in order to gain greater insight into these interactions and the experience and impact of fatigue. This mixed-methods study aimed to explore the experience and impact of fatigue in adults with pAPS.

## Methods

### Study design

This mixed-methods study adopted a sequential, explanatory design informed by the priority sequence model.^28,29^ In this model, an initial decision about the priority of a quantitative or qualitative method is taken. Next, the sequence determines whether the complementary method serves either as a preliminary or a follow-up phase. For this study, a cross-sectional survey was conducted to investigate relationships between fatigue and key psychosocial variables. Then, follow-up qualitative data were collected to explore the interactions identified in the quantitative phase and the direct experience and impact of fatigue in people with pAPS.

The study gained governance approval from North of Scotland Research Ethics Committee (14/NS/0026, 02/07/2014) and Research and Innovation from Guy’s and St Thomas’ Hospital NHS Foundation Trust (12/03/2015).

### Quantitative phase

#### Participants and data collection

Patients were eligible to be enrolled onto the study if they were aged ≥18 years with pAPS (Sydney classification criteria^30^) for six months or more and had adequate verbal and written English language. Patients who had other autoimmune rheumatic or inflammatory co-morbid conditions, malignancy, active chronic infections, current alcohol and/or drug abuse or dependence, a body mass index  > 30 kg/m^2^ recorded in their medical records and/or were pregnant or breastfeeding were excluded. Patients with positive ANA were also excluded, as this may indicate APS secondary to other RMDs, such as SLE.^31,32^ Members of the direct care team reviewed the medical records of patients attending routine clinical appointments at a tertiary health-care centre for the management of APS in the UK. Potentially suitable patients were identified and approached by the members of the direct care team to gauge their interest in the study and to confirm their eligibility. Interested patients received a questionnaire pack, which comprised study information, a consent form and six questionnaires to self-complete after their appointment. Alternatively, patients completed the questionnaires at home and returned them to the researchers in a prepaid envelope. A researcher offered to support questionnaire completion after the clinical appointment or via telephone at a mutually convenient time. Patients who did not return the questionnaires were sent a second pack four to six weeks later. No further reminders were issued to non-responders.

#### Sample size

Using G*Power v3.1.9.2, a medium effect (*f*^2^ = 0.15, *R*^2^ = 0.13 equivalent) in variance of FS with *p* = 0.05, a power of 0.80, and including four predictor variables required 89 participants.

#### Sociodemographic and disease characteristics

Sociodemographic and disease characteristics, including age, sex, birthplace, ethnicity, employment status (full-time, part-time, retired, unemployed, higher education or other), disability registration (yes/no) and duration of APS, were collected using a bespoke, self-administered questionnaire.

#### Variables

Participants completed five self-completed, validated and standardised questionnaires.

#### Fatigue

Fatigue over the past week was measured using the 13-item Functional Assessment of Chronic Illness Therapy (FACIT)-Fatigue Scale (FS; a four-point Likert scale ranging from ‘not at all fatigued’ to ’very much fatigued’), with a lower score indicating greater fatigue (range 0–52). A score of <30 indicates severe fatigue.^33,34^

#### Mood

Mood was assessed with the nine-item Patient Health Questionnaire (PHQ-9; a four-point Likert scale ranging from ‘not at all’ to ’nearly every day’) which is designed to correspond to the diagnosis of depression. A higher score indicates lower mood/depression (range 0–27), and scores ≥10 represent clinically depressive symptoms.^35,36^

#### Physical activity

Physical activity over the preceding seven days was measured with the four-item International Physical Activity Questionnaire – short form (IPAQ). The metabolic equivalent of task was calculated over seven days (METS/minutes/week – IPAQMETS), and scores ≥600 MET/minutes/week were considered physically active.^37^

#### Sleep quality

The quality of sleep over the past month was assessed using the seven-component 19-item Pittsburgh Sleep Quality Index (PSQI; four-point Likert scale ranging from ‘not during the past month’ to ’three or more times a week’). A higher total PSQI score (range 0–21) represents lower sleep quality. A total PSQI score of  > 5 represents severe difficulties in at least two components.^38,39^

#### Perceived social support

Perceived social support from family, friends and significant others was evaluated via the 12-item Multi-Dimensional Perceived Social Support Scale (MSPSS; seven-point Likert scale ranging from ‘strongly disagree’ to ‘strongly agree’). Higher scores indicate higher perceived social support (1–2.9, low; 3–5, moderate; 5.1–7, high).^40,41^

### Qualitative phase

A purposive subsample of participants enrolled in the cross-sectional survey was interviewed by one of two researchers (J.A. or S.G.).^42^ The interviewees varied in relation to most sociodemographic characteristics, APS type and lived experiences.

Interviewers were not directly involved in participants’ health care and were supervised by an experienced qualitative researcher (H.L.). The audio-recorded interviews were conducted either face to face or by telephone, subject to each participant’s preference. A topic guide was developed a priori derived from other RMD fatigue literature^8,9,11^ and refined with researchers’ and patients’ feedback to include questions aligned to the key quantitative variables. The semi-structured interview schedule allowed the interviewer to ask open-ended questions so that the interviewee could diverge or expand the experiences or situations in more detail.^43^ A pilot study was completed with three participants to assess comprehension and relevance of questions, timing and subjectively perceived research burden. No further changes were necessary to the interview guide (Table 1). Subtle judgement between interviewers and the supervisor led to recruitment termination when data saturation of themes had been reached (i.e. no new information was reported by the participants).^44^

**Table 1.** Interview topic guide to explore the experience of fatigue in adults with primary antiphospholipid syndrome (pAPS).

1. Do you ever feel fatigued?
2. How do you feel about moving around and being active?
3. Tell me about your experiences of fatigue? Can you describe it to me?
4. What do you think causes your fatigue?
5. How do the symptoms of your fatigue vary?
6. Does fatigue impact on your life? How?
7. How do you feel about your personal level of physical activity/exercise?
8. Do you manage to integrate physical activity/exercise in your daily routine?
9. Do you talk to other people about your fatigue/activity levels?
10. What is your experience of talking to doctors and other healthcare professionals about your fatigue?
11. What is your experience of talking to doctors and other healthcare professionals about your physical activity/exercise?
12. Have doctors or other healthcare professionals suggested physical activity/exercise as a way of managing fatigue?

### Data analysis

#### Statistical analysis

Analyses were completed using IBM SPSS Statistics for Windows v25.0 (IBM Corp., Armonk, NY). Statistical significance was set at *p* < 0.05. Frequencies and mean (standard deviation (*SD*)) values were reported for categorical and continuous descriptive variables, respectively. Relationships with FS (criterion variable) were explored using two-tailed Spearman’s rho correlation coefficients. A backward stepwise regression was performed including all measures associated with FS at *p* < 0.05 in the bivariate analyses to determine whether they predicted the FS score. The model with the largest adjusted *R*^2^ model was used to perform a multiple linear regression analysis. Missing values were excluded pairwise. Univariate (Studentised residual values±3*SD*) and multivariate outliers (Mahalanobis distance *p* < 0.01) were excluded, and models were evaluated for multicollinearity, normal and independent errors and homoscedasticity.

#### Qualitative data analysis

The interviews were anonymised and transcribed verbatim by one researcher and one professional transcribing agency, and the text was analysed by two researchers (J.A. and H.L.) using the principles of Framework Analysis^45^ via a five-stage structured approach^46^ that included (a) familiarisation with text, (b) coding within the qualitative computer package NVIVO v10 (QSR International Pty Ltd),^47^ (c) building categories and themes, (d) identification of a thematic framework and (e) linking findings with theoretical concepts.

Validation of data included (a) checking initial codes with one external researcher from one transcript, (b) presentation of initial findings from the pilot study to the supervisor, (c) discussing emerging themes from two additional transcripts and (d) single counting. Transcript accuracy was checked by the participants against original recordings. A balanced reporting of data is a recognized validation strategy.^47^ Therefore, a range of accounts are presented when available.

## Results: quantitative phase

### Descriptive statistics

A total of 105 potentially eligible patients were approached between July 2014 and June 2017. Two people with communication difficulties (one with a hearing impairment and one with insufficient English) were excluded. A total of 103 participants were therefore enrolled onto the study (18 males, *M*_age_ = 50.3 years, *SD* = 10.1 years). Data from 16 participants (10 male, *M*_age_ = 49.7 years, *SD* = 10.1 years) were omitted due to incomplete data. Therefore, 87 participants (8 male, *M*_age_ = 50.3 years, *SD* = 10.4 years) were included in the analysis (Table 2). A total of 62% of participants reported severe fatigue, 40% reported depression, 24% were classified as physically inactive, 69% revealed severe sleep difficulties and 35% reported low-moderate levels of perceived social support.

**Table 2.** Sociodemographic and clinical characteristics of adults with pAPS.

	Cross-sectional survey respondents	Interview participants
	(*N* = 87)	(*N* = 20)
Sex (female), *n* (%)	79.0 (90.8)	20.0 (100)
Age (years)	50.3 (10.4)	49.3 (9.7)
Disease duration (months)	143.0 (92.0)	149.4 (97.5)
Ethnicity (White British), *n* (%)	75.0 (86.0)	18.0 (90.0)
Registered disabled (yes), *n* (%)	13.0 (14.0)	5.0 (25.0)
Employment status		
Full time, *n* (%)	31.0 (36.0)	6.0 (30.0)
Part time, *n* (%)	16.0 (18.0)	7.0 (35.0)
Retired, *n* (%)	15.0 (17.0)	2.0 (10.0)
Unemployed, *n* (%)	13.0 (15.0)	2.0 (10)
Higher education, *n* (%)	2.0 (2.0)	0.0 (0.0)
Other, *n* (%)	10.0 (12.0)	3.0 (15.0)
FS	25.7 (13.0)^b^	24.0 (11.5)
PHQ-9	9.5 (6.6)	9.4 (5.2)
IPAQMETs	2982.4 (3433.8)^a^	2236.3 (2540.2)
PSQI	8.8 (4.6)^c^	9.3 (3.9)
MSPSS	5.4 (1.4)^a^	5.2 (1.7)

Values shown are mean (standard deviation) unless otherwise indicated.

^a^*N* = 85; ^b^*N* = 84 ^c^*N* = 71.

FACIT: F Fatigue subscale; IPAQMETs: International Physical Activity Questionnaire (Metabolic Equivalent of Task/minutes/week); MSPSS: The Multi-Dimensional Perceived Social Support Scale; PHQ-9: Patient Health Questionnaire; PSQI: Pittsburgh Sleep Quality Index.

### Bivariate associations with fatigue

FS was negatively associated with PHQ9 (*r*_s_(84) = –0.750, *p* < 0.001) and PSQI (*r*_s_(79) = –0.515, *p* < 0.001), and positively associated with IPAQMETS (*r*_s_(81) = 0.232, *p* = 0.037) and MSPSS (*r*_s_(82) = 0.239, *p* = 0.030; Table 3).

**Table 3:** Descriptive statistics and bivariate correlations between fatigue, mood, physical activity, sleep quality and social support in adults with pAPS.

	FS	PHQ-9	IPAQMETs	PSQI	MSPSS
FS	1	–0.750**	0.232*	–0.515**	0.239*
PHQ-9		1	–0.105	0.613**	–0.238*
IPAQMETs			1	–0.098	0.018
PSQI				1	–0.071
MSPSS					1

*p < 0.05; **p < 0.01.

### Multivariate analyses

The backward stepwise regression included all variables (Table 4). The final best-fit model explained 56% of the variance in FS (adjusted *R*^2^ = 0.560, *F*(3, 74) = 33.65, *p* < 0.001) and included PHQ9 and IPAQMETS as significant predictors and PSQI as a non-significant predictor but not MSPSS (Table 5). Unstandardized beta coefficients indicated that for every unit increase in PHQ9 scores, FS decreased by 1.336 points (95% confidence interval (CI) –1.72 to –0.95, SE *B* = 0.19, β = –0.67, *p* < 0.001), and for every unit increase in IPAQMETS, FS increased by 0.001 points (95% CI 0.00004–0.00119, SE *B* = 0.0003, β = 0.15, *p* = 0.037). For every unit increase in PSQI scores, FS decreased by 0.286 points (95% CI –0.84 to 0.27, SE *B* = 0.28, β = –0.1, *p* = 0.306).

**Table 4.** Backward multiple linear regression models for predicting fatigue (FS) in adults with pAPS.

Model	R	*R* ^2^	Adjusted *R*^2^	SE of the estimate	F	p
Baseline model	0.762^a^	0.581	0.558	8.637	*F*(4, 73) = 25.33	<0.001
Best-fit model	0.760^b^	0.577	0.560	8.622	*F*(3, 74) = 33.65	<0.001

^a^Predictors: IPAQMETS, PHQ-9, MSPSS, PSQI.

^b^Predictors: IPAQMETS, PHQ-9, PSQI.

**Table 5.** Beta coefficients for the baseline model and best fit model, for predicting fatigue (FS) in adults with pAPS.

		Unstandardized coefficients		Standardized coefficients			95% confidence interval for B	
		B	Std. Error	β	t	p	Lower bound	Upper bound
Baseline model	PHQ-9	–1.297	0.197	–0.655	–6.567	<0.001	–1.690	–0.903
	IPAQMETs	0.001	<0.001	0.159	2.085	0.041	<0.001	0.001
	PSQI	–0.317	0.280	–0.111	–1.132	0.262	–0.876	0.241
	MSPSS	0.616	0.716	0.067	0.861	0.392	–0.810	2.042
Best-fit model	PHQ-9	–1.336	0.192	–0.674	–6.966	<0.001	–1.718	–0.954
** **	IPAQMETS	0.001	<0.001	0.162	2.127	0.037	<0.001	0.001
	PSQI	–0.286	0.278	–0.100	–1.032	0.306	–0.839	0.267

## Results: qualitative phase

Twenty consenting participants were interviewed (duration: 20–60 minutes). The participants were all female and predominantly white (Table 2). Three key themes were identified: (a) characteristics of fatigue, (b) impact on life and (c) coping strategies, with two to four linked subthemes.

### Theme 1: characteristics of fatigue

#### Use of metaphors

All participants expressed experiencing fatigue at some time and used a range of metaphors to describe their fatigue. For example: ‘fatigue hits you like a truck’ (P7), ‘I feel like a rag doll with no stuffing left’ (P1), ‘I’m climbing up a really big hill with a massive rucksack on my back, and I’m struggling to get to the top’ (P12) and ‘[fatigue is] almost like a physical weight pulling me down feeling’ (P19).

#### Unpredictability and fluctuation of fatigue

Our participants (14/20) described that fatigue was unpredictable and variable and therefore interfered with their daily activities of living. They were uncertain about the causes or triggers for their fatigue. For example: ‘You know, the fatigue like I said is, it can happen any day and come out of the blue’ (P15) and ‘So it’s very variable [fatigue], and it’s not even the fact that I’ve overdone it the day before, which sometimes can help bring on the fatigue even more, but sometimes, umm, I’ve been absolutely fine, just been doing a steady pace for days, then boom it suddenly hits me and I’m in bed most of the day’ (P13).

#### Severity of fatigue

Many participants (17/20) commented on the severity of their fatigue and tended to rate this using a numerical scale or labels, such as ‘mild’, ‘manageable’, ‘bad ‘and ‘unbearable’ to convey the gravity of the fatigue. For example: ‘I think my fatigue is, on a scale of 1 to 10, 5 at the most, 3 probably most of the time’ (P12) and ‘I don’t know [how many times a week]. I have a couple really bad days a week’ (P19).

### Theme 2: impact on life

The impact of fatigue on each individual’s life was personal and wide ranging. All participants reported ‘low motivation’ or ‘forcing themselves’ to carry on with everyday life during periods of fatigue. Participants described the huge effort required to get through days when they were experiencing overwhelming fatigue and lack of energy.

#### Physical activity

Most participants (16/20) regarded themselves as physically active in their private and/or work lives. However, the frequency, intensity, duration and format of physical activity varied between interviewees. They were aware of the need to be active for their general health and were broadly aware of the public-health recommendations. For example: ‘I do, roughly, you know, between 15 and 20 minutes of walking a day … which is what the national, you know, erm … guidelines say’ (P15). Most participants (16/20) described a relationship between physical activity and fatigue levels but noted that at times, fatigue was a barrier to completing physical activity. For example: ‘Sometimes it [my physical activity] will be morning, sometimes it will be the afternoon and sometimes early evening, it really depends. But usually sort of around one-ish … I have to walk the dog’ (P6) and ‘…when I did have bad fatigue periods, I couldn’t swim for a few weeks’ (P10). Some participants (7/20) observed that exercise was a helpful management strategy for fatigue, at times. For example: ‘…sometimes exercise can help fatigue, but I also know, and I can read my body quite well, when sometimes it is going to make me worse’ (P11) and ‘…if I’m sedentary, it just makes it [fatigue] worse’ (P14).

#### Quality of sleep

Some participants (5/20) reported that sleep quality influenced their fatigue and that this was difficult to manage and frustrating. For example: ‘A poor night’s sleep, makes me, it sort of extenuates tiredness, and it feels worse, and when my INR [International Normalised Ratio] is low’ (P14) and ‘I hate it when I am so tired, for instance recently I was on holiday with a friend and I was determined not to do it [have a hour sleep during the day], but I’m afraid I did have to do it’ (P7).

#### Impact of mood

Some participants (8/20) described how mood and negative thoughts affected their fatigue. These thoughts could be intrusive and unhelpful, and this influenced their ability to accomplish their daily activities. For example:

…in some ways my mood can affect my fatigue, but it’s making me go more the other way at the moment, it’s making me feel like I can’t sleep … I’m constantly thinking about things that are upsetting me or negative thoughts … and of course my body is getting tired, so by the time two days later I’m feeling more fatigued than I would have done if I hadn’t been feeling so anxious and agitated. (P15)

A few participants (3/20) also reported that fatigue also influenced their mood.

…I do believe that fatigue can trigger in me, slight depression, I’m not depressed by nature, but obviously the energy and dealing with it day in and day out when it [fatigue] is going on it is tiresome, and I find that your mind-set is not so clear, and you are not as rational as you would be, if you feel refreshed and positive. (P11)

#### Impact on work

There was no doubt that fatigue impacted upon participants’ salaried or daily work, which meant they had to make adaptations and were feeling unable to carry out complex tasks at times. For example: ‘[I] work flexi-time, so on a day where I just think I cannot do this anymore, I just need to get myself home, I can leave within reason’ (P10) and ‘…I only work part time, whether I would be able to work full-time? I don’t know the answer to that. Maybe I would get too fatigued if I worked full time’ (P6).

### Theme 3: coping strategies

All participants (20/20) employed a range of coping strategies to manage their fatigue. The strategies selected depended on the severity of the fatigue and were often informed by past successful experiences of dealing with their fatigue.

#### Individual coping strategies

Most participants (16/20) described how they applied individual coping strategies in their personal life, and pacing was a popular strategy. For example: ‘If I’m doing something that needs doing, and I start to get it [fatigue], I just carry on. I will suffer for it afterwards, but I don’t want it [fatigue] to take over my life’ (P1) and ‘I think you have to pace, you have to be careful and you have to pace yourself, you can’t do this, that and the other, and so, but even when you pace yourself you still get those times [of fatigue], for no reason, and you look at it and go, oh, it is like that today is it?’ (P12).

#### Support from healthcare professionals

Many participants (19/20) had discussed fatigue with a healthcare professional. However, the topic of fatigue often received a mixed response by clinicians and tended not to be routinely reviewed. For example:

I did obviously talk about it [fatigue] when I was first diagnosed, and they [clinicians] did take it seriously, but now there is no point keeping going on about it to them [clinicians]. (P7)

When I spoke to my GP, my GP said ‘Well, you know more about this than I do’. So, I stopped at that point. But, although having said that, the rheumatologists that I saw when I had the problem with the steroids, he understood completely about the fatigue. Because he actually said to me, I told him about my symptoms with the joints in the muscles and things, he said to me, ‘Oh, you must get so tired’, and I said, ‘Well, it’s wonderful to hear someone actually say that I should be getting tired!’. (P7)

## Discussion

This mixed-methods study is one of the first to explore the experience and impact of fatigue in adults with pAPS. The findings showed that fatigue was common, unpredictable and sometimes overwhelming in people with pAPS. It was influenced by factors such as low mood and physical inactivity. Participants had developed their own coping strategies to manage their fatigue and generally reported receiving high levels of social support. They reported minimal or variable understanding from health-care professionals.

Almost two thirds of our participants reported experiencing severe fatigue at times. Participants were vigilant about monitoring their fatigue so they could adopt strategies to mitigate the symptom. However, they found fatigue challenging to manage and difficult to explain to others, including health-care professionals. Consequently, participants used metaphors to express the character of fatigue and convey their increased effort and loss of energy, similar to people with long-term conditions such as SLE^9^ or RA.^11^

More than a third of our participants reported low mood, and this was associated with higher fatigue levels. The relationship between mood and fatigue is well described in people with other long-term conditions,^16,48^ and mood predicts fatigue, even after adjustment for pain and other potentially confounding variables in some RMDs.^20^ Low mood can affect sleep, and many of our participants reported sleep difficulties like people with other RMDs.^15,17,20^ Sleep disturbances could be due to pain, stress or influenced by medication.^17^ Whilst many interviewees described a clear link between fatigue and sleep, sleep quality did not independently predict fatigue in our quantitative data. This may be because sleep quality has an indirect effect on fatigue and mediates the direct influence of depression or physical inactivity on fatigue.^15,17^

More than three quarters of our respondents reported meeting physical activity recommendations, which is higher than people with other RMDs.^22,23,49^ This may be due to over-reporting because these data were collected using self-completed questionnaires, which is subject to recall bias. Alternatively, since many participants were employed or had active caring responsibilities, this finding may reflect activity undertaken for transportation (i.e. walking) related to their daily work. This study showed that greater physical activity was directly related to lower fatigue, and some interviewees stated that physical activity/exercise was a helpful management strategy for fatigue. Brief home-based physical activity and exercise interventions improve fatigue and sleep quality in people with RA. So, this may be a promising intervention approach for people with pAPS.^50,51^ However, some interviewees found the physical activity hard to complete due to the unpredictability of fatigue. So, tailored strategies or interventions may be needed to accommodate fluctuations in fatigue.

Participants developed their own coping strategies, particularly pacing, to manage their fatigue. They mostly received good social support from family, friends and significant others (i.e. perceived emotional support), concurring with other research,^52^ and it is proposed that social support influences self-efficacy and has a buffering effect on fatigue.^20,26^ Whilst social support correlated with fatigue in our study, it did not independently explain the variance in fatigue in our multivariate analyses and exploration of the other types of perceived social support (i.e. instrumental (practical support such as housework) or informational (education) support) may clarify this relationship.^52^ Informational social support could be provided by clinicians, although our participants commented that fatigue was not regularly discussed in consultations. This could be because clinicians do not recognise the presence or impact of fatigue in people with pAPS or because it is not acknowledged in management recommendations.^27^ Alternatively, the lack of evidence-based management strategies for fatigue in pAPS may mean that it is rarely a treatment focus. A multidisciplinary team approach may optimise the management of pAPS, similar to other RMDs such as RA.^53–55^

The conceptual model of fatigue for patients with RA by Hewlett et al. aligns with our findings.^14^ Factors such as pAPS itself, cognitive and behavioural dimensions (e.g. mood, physical activity) and personal aspects (e.g. social support) play a dynamic role in the experiences of fatigue and how people cope with it. This model may help researchers, clinicians and patients clarify the focus for future interventions and measurements to inform treatment and self-management.

This study has several strengths. The mixed-methodology approach facilitated the integration of the findings from both phases within a single study. The findings are complementary and provide a deeper insight into the experience of fatigue in adults with pAPS.^28,29^ Our eligibility criteria were robust, and adults with co-morbidities known to influence fatigue (e.g. obesity) were excluded to minimise confounding factors. The number of medical records reviewed and reasons for patients’ ineligibility following review of medical notes were not documented. However, the team enrolled a range of participants with pAPS and only two potentially suitable patients, who were identified and approached by the clinic team, were excluded. Our cohort reflected the female predominance which exists in pAPS. However, patients were recruited from a single centre, there was relatively limited ethnic and sex diversity in our study population and the educational status of the participants was not recorded.

Our survey explored key factors known to influence fatigue in other RMDs, but other aspects may contribute to fatigue (e.g. physical capacity, end organ damage), and these require investigation. As data from our quantitative phase was cross-sectional, no causality can be inferred from the findings, and the reciprocal effects of fatigue on other features cannot be observed (e.g. greater fatigue may lead to lower mood or less activity).

Fatigue is common and impacts the lives of adults with pAPS. Enhancing communication between patients and clinicians so that symptoms which are important to patients, such as fatigue, are identified and addressed appropriately is crucial to optimise the management of pAPS. Developing effective multidisciplinary interventions to support self-management of fatigue with patients, clinicians and researchers is vital.

## References

[1] Cervera R Serrano R Pons-Estel GJ, et al Morbidity and mortality in the antiphospholipid syndrome during a 10-year period: a multicentre prospective study of 1000 patients. Ann Rheum Dis 2015; 74: 1011–1018. 24464962 10.1136/annrheumdis-2013-204838

[2] Myers B Pavord S. Diagnosis and management of antiphospholipid syndrome in pregnancy. Obstet Gynaecol 2011; 13: 15–21.

[3] Durcan L Petri M. Epidemiology of the antiphospholipid syndrome In: R Cervera JC Reverter M Khamashta (eds) Handbook of systemic autoimmune diseases. Amsterdam, Netherlands: Elsevier, 2017, pp.17–30.

[4] Jallow T D’Cruz D Lempp H. Antiphospholipid syndrome. BMJ 2015; 350: h1426. 25838412 10.1136/bmj.h1426

[5] Georgopoulou S Efraimidou S MacLennon SJ Ibrahim F Cox T. Antiphospholipid (Hughes) syndrome: description of population and health-related quality of life (HRQoL) using the SF-36. Lupus 2015; 24: 174–179. 25239925 10.1177/0961203314551809

[6] Piper BF. Pathophysiological phenomena in nursing: human responses to illness. Philadelphia, PA: WB Saunders, 1993.

[7] Norheim KB Jonsson G Omdal R. Biological mechanisms of chronic fatigue. Rheumatology 2011; 50: 1009–1018. 21285230 10.1093/rheumatology/keq454

[8] Hewlett S Cockshott Z Byron M Tipler S Pope D Hehir M. Patients’ perceptions of fatigue in rheumatoid arthritis: overwhelming, uncontrollable, ignored. Arthitis Rheum 2005; 53: 697–702. 10.1002/art.21450 16208668

[9] Pettersson S Möller S Svenungsson E Gunnarsson I Welin Henriksson E. Women’s experience of SLE-related fatigue: a focus group interview study. Rheumatology 2010; 49: 1935–1942. 20573692 10.1093/rheumatology/keq174

[10] Eilertsen G Ormstad H Kirkevold M Mengshoel AM Söderberg S Olsson M. Similarities and differences in the experience of fatigue among people living with fibromyalgia, multiple sclerosis, ankylosing spondylitis and stroke. J Clin Nurs 2015; 24: 2023–2034. 25661994 10.1111/jocn.12774

[11] Primdahl J Hegelund A Lorenzen AG Loeppenthin K Dures E Appel Esbensen B. The experience of people with rheumatoid arthritis living with fatigue: a qualitative metasynthesis. BMJ Open 2019; 9: e024338. 10.1136/bmjopen-2018-024338 PMC6475175 30898808

[12] Seifert O Baerwald C. Impact of fatigue on rheumatic diseases. Best Pract Res Clin Rheumatol 2019; 33: 101435. 31703791 10.1016/j.berh.2019.101435

[13] Wolfe F Hawley DJ Wilson K. The prevalence and meaning of fatigue in rheumatic disease. J Rheumatol 1996; 23: 1407–1417. 8856621

[14] Hewlett S Chalder T Choy E, et al Fatigue in rheumatoid arthritis: time for a conceptual model. Rheumatology (Oxford) 2011; 50: 1004–1006. 20819797 10.1093/rheumatology/keq282

[15] Katz P Margaretten M Trupin L Schmajuk G Yazdany J Yelin E. Role of sleep disturbance, depression, obesity, and physical inactivity in fatigue in rheumatoid arthritis. Arthritis Care Res (Hoboken) 2016; 68: 81–90. 25779719 10.1002/acr.22577 PMC6083443

[16] Matcham F Ali S Hotopf M Chalder T. Psychological correlates of fatigue in rheumatoid arthritis: a systematic review. Clin Psychol Rev 2015; 39: 16–29. 25912978 10.1016/j.cpr.2015.03.004

[17] Ahn GE Ramsey-Goldman R. Fatigue in systemic lupus erythematosus. Int J Clin Rheumatol 2012; 7: 217–227. 10.2217/IJR.12.4 PMC3380630 22737181

[18] Matcham F Rayner L Steer S Hotopf M. The prevalence of depression in rheumatoid arthritis: a systematic review and meta-analysis. Rheumatology (Oxford) 2013; 52: 2136–2148. 24003249 10.1093/rheumatology/ket169 PMC3828510

[19] Figueiredo-Braga M Cornaby C Cortez A, et al, Depression and anxiety in systemic lupus erythematosus: The crosstalk between immunological, clinical, and psychosocial factors. Medicine (Baltimore) 2018; 97: e11376. 29995777 10.1097/MD.0000000000011376 PMC6076116

[20] Nikolaus S Bode C Taal E Van De Laar MA, et al Fatigue and factors related to fatigue in rheumatoid arthritis: a systematic review. Arthritis Care Res (Hoboken) 2013; 65: 1128–1146. 23335492 10.1002/acr.21949

[21] Palagini L Tani C Bruno RM, et al Poor sleep quality in systemic lupus erythematosus: does it depend on depressive symptoms? Lupus 2014; 23: 1350–1357. 24944187 10.1177/0961203314540762

[22] Manning VL Hurley MV Scott DL Bearne LM. Are patients meeting the updated physical activity guidelines? Physical activity participation, recommendation, and preferences among inner-city adults with rheumatic diseases. J Clin Rheumatol 2012; 18: 399–404. 23188205 10.1097/RHU.0b013e3182779cb6

[23] Sokka T Häkkinen A Kautiainen H, et al Physical inactivity in patients with rheumatoid arthritis: data from twenty-one countries in a cross-sectional, international study. Arthritis Rheum 2008; 59: 42–50. 18163412 10.1002/art.23255

[24] Mahieu MA Ahn GE Chmiel JS, et al Fatigue, patient reported outcomes, and objective measurement of physical activity in systemic lupus erythematosus. Lupus 2016; 25: 1190–1199. 26869353 10.1177/0961203316631632 PMC4980272

[25] Katz P. Causes and consequences of fatigue in rheumatoid arthritis. Curr Opin Rheumatol 2017; 29: 269–276. 28207494 10.1097/BOR.0000000000000376

[26] Jump RL Robinson ME Armstrong AE Barnes EV Kilbourn KM Richards HB. Fatigue in systemic lupus erythematosus: contributions of disease activity, pain, depression, and perceived social support. J Rheumatol 2005; 32: 1699–1705. 16142863

[27] Tektonidou MG Andreoli L Limper M, et al EULAR recommendations for the management of antiphospholipid syndrome in adults. Ann Rheum Dis 2019; 78: 1296–1304. 31092409 10.1136/annrheumdis-2019-215213 PMC11034817

[28] Creswell JW Plano Clark VL. Designing and conducting mixed methods research. 3rd ed Los Angeles, CA: Sage, 2017.

[29] Morgan DL. Practical strategies for combining qualitative and quantitative methods: application for health research. Qual Health Res 1998; 8: 362–376. 10558337 10.1177/104973239800800307

[30] Miyakis S Lockshin MD Atsumi T, et al International consensus statement on an update of the classification criteria for definite antiphospholipid syndrome (APS). J Thromb Haemost 2006; 4: 295–306. 16420554 10.1111/j.1538-7836.2006.01753.x

[31] Freire PV Watanabe E Dos Santos NR Bueno C Bonfá E De Carvalho JF, et al Distinct antibody profile: a clue to primary antiphospholipid syndrome evolving into systemic lupus erythematosus? Clin Rheumatol 2014; 33: 349–353. 24420722 10.1007/s10067-013-2472-3

[32] Asherson RA Cervera R Piette JC Shoenfeld Y (eds). The antiphospholipid syndrome. Boca Raton, FL: CRC Press, 2018.

[33] Webster K Cella D Yost K. The Functional Assessment of Chronic Illness Therapy (FACIT) measurement system: properties, applications, and interpretation. Health Qual Life Outcomes 2003; 1: 79. 14678568 10.1186/1477-7525-1-79 PMC317391

[34] Lai J-S Beaumont JL Ogale S, et al Validation of the functional assessment of chronic illness therapy-fatigue scale in patients with moderately to severely active systemic lupus erythematosus, participating in a clinical trial. J Rheumatol 2011; 38: 672–679. 21239746 10.3899/jrheum.100799

[35] Kroenke K Spitzer RL Williams JB. The PHQ-9: validity of a brief depression severity measure. J Gen Intern Med 2001; 16: 606–613. 11556941 10.1046/j.1525-1497.2001.016009606.x PMC1495268

[36] Kroenke K Spitzer RL. The PHQ-9: a new depression diagnostic and severity measure. Psychiatr Ann 2002; 32: 509–515.

[37] Craig C Marshall AL Sjöström M, et al International Physical Activity Questionnaire (IPAQ): 12-country reliability and validity. Med Sci Sports Exerc 2003; 35: 81–95. 10.1249/01.MSS.0000078924.61453.FB 12900694

[38] Buysse DJ Reynolds CF 3rd Monk TH Berman SR Kupfer DJ. The Pittsburgh Sleep Quality Index: a new instrument for psychiatric practice and research. Psychiatry Res 1989; 28:193–213. 2748771 10.1016/0165-1781(89)90047-4

[39] Omachi TA. Measures of sleep in rheumatologic diseases: Epworth Sleepiness Scale, functional Outcome of Sleep Questionnaire, Insomnia Severity Index and Pittsburgh Sleep Quality Index. Arthritis Care Res (Hoboken) 2011; 63: S287–296. 22588751 10.1002/acr.20544 PMC3711174

[40] Zimet GD Dahlem NW Zimet SG Farley GK. The Multidimensional Scale of Perceived Social Support. J Pers Assess 1988; 52: 30–41.

[41] Zimet GD Powell SS Farley GK Werkman S Berkoff KA. Psychometric characteristics of the Multidimensional Scale of Perceived Social Support. J Pers Assess 1990; 55: 610–617. 2280326 10.1080/00223891.1990.9674095

[42] Palinkas L Horwitz SM Green CA Wisdom JP Duan N Hoagwood K. Purposeful sampling for qualitative data collection and analysis in mixed method implementation research. Adm Policy Ment Health 2015; 42: 533–544. 24193818 10.1007/s10488-013-0528-y PMC4012002

[43] Britten N. Qualitative research: qualitative interviews in medical research. BMJ 1995; 311: 251–253. 7627048 10.1136/bmj.311.6999.251 PMC2550292

[44] Guest G Bunce A Johnson L. How many interviews are enough? An experiment with data saturation and variability. Family Health Int 2006; 18: 59–82.

[45] Ritchie J Spencer L. Qualitative data analysis for applied policy research In A Bryman RG Burgess (eds). Analysing qualitative data. London: Routledge, 1994, pp.173–194.

[46] Braun V Clarke V. Using thematic analysis in psychology. Qual Res Psychol 2006; 3: 77–101.

[47] Seale C. Using computers to analyse qualitative data In D Silverman (ed). Doing qualitative research: a practical handbook. London: Sage, 1999, pp.154–172.

[48] Arnaud L Gavand PE Voll R, et al Predictors of fatigue and severe fatigue in a large international cohort of patients with systemic lupus erythematosus and a systematic review of the literature. Rheumatology 2019; 58: 987–996. 30597077 10.1093/rheumatology/key398

[49] Loppenthin K Esbensen BA Østergaard M, et al Physical activity and the association with fatigue and sleep in Danish patients with rheumatoid arthritis. Rheumatol Int 2015; 35: 1655–1664. 25947325 10.1007/s00296-015-3274-5

[50] Durcan L Wilson F Cunnane G. The effect of exercise on sleep and fatigue in rheumatoid arthritis: a randomized controlled study. J Rheumatol 2014; 41: 1966–1973. 25128510 10.3899/jrheum.131282

[51] Katz P Margaretten M Gregorich S Trupin L, Physical activity to reduce fatigue in rheumatoid arthritis: a randomized controlled trial. Arthritis Care Res (Hoboken) 2018; 70: 1–10. 28378441 10.1002/acr.23230

[52] Georgopoulou S Efraimidou S MacLennan SJ Ibrahim F Cox T. The relationship between social support and health-related quality of life in patients with antiphospholipid (hughes) syndrome. Mod Rheumatol 2018; 28:147–155. 28463088 10.1080/14397595.2017.1317319

[53] Bearne LM Byrne AM Segrave H White CM. Multidisciplinary team care for people with rheumatoid arthritis: a systematic review and meta-analysis. Rheumatol Int 2016; 36:311–324. 26563338 10.1007/s00296-015-3380-4

[54] McCuish W Bearne LM. Do inpatient multidisciplinary rehabilitation programmes improve health status in people with long-term musculoskeletal conditions? A service evaluation. Musculoskeletal Care 2014; 12: 244–250. 24840767 10.1002/msc.1072

[55] National Institute for Health and Care Excellence. Rheumatoid arthritis in adults: management, www.nice.org.uk/guidance/cg79 (2015, accessed 21 May 2016).

